# A “scattered” SCAT in a football goalkeeper: a case report

**DOI:** 10.17159/2078-516X/2020/v32i1a7737

**Published:** 2020-01-01

**Authors:** S Bosch, PL Viviers, R de Villiers, W Derman

**Affiliations:** 1 Institute of Sport and Exercise Medicine, Division of Orthopaedics, Faculty of Medicine and Health Sciences, Stellenbosch University, South Africa; 2 IOC Research Centre, Cape Town, South Africa; 3 FIFA Medical Centre of Excellence, Stellenbosch University, South Africa; 4 Campus Health Service, Stellenbosch University, South Africa; 5 Catholic University of Leuven, Department of Physical Medicine and Rehabilitation, Leuven, Belgium; 6 Winelands Radiology, Institute of Orthopaedics and Rheumatology, Stellenbosch, South Africa

**Keywords:** concussion, soccer, sports-related head injury

## Abstract

**Background:**

In an acute field-side setting, it is often challenging to differentiate benign sports-related concussion (SRC) from potential, more sinister, intracranial pathology. Moreover, recovery in the ensuing days and weeks is often complex as the resolution of classical signs and symptoms does not always follow a standard pattern.

**Aim:**

To highlight the value of a structured and repeated thorough clinical assessment approach toward SRC, particularly as atypical and unexpected sequences in patient recovery patterns may require further specialist referral and intervention.

**Findings:**

A football goalkeeper sustained a concussion in which symptoms failed to resolve as expected. Deterioration in his clinical condition led to an eventual diagnosis of Chiari malformation (type I), which required surgical intervention.

**Implications:**

Non-typical recovery patterns of concussion may be indicative of increased severity when considered retrospectively. However, clinicians should not discount the possibility of underlying conditions.

## Case report

A 20 year old male football goalkeeper sustained a traumatic head injury due to a collision with another player while reaching for the ball. Upon impact, he was elbowed on the right mastoid and collapsed, but got up immediately after the incident. He felt disoriented and confused and was unable to prevent a goal from being scored seconds after the impact. He was subsequently removed from the field of play. There was no loss of consciousness at any time. During the sideline evaluation, he complained of dizziness, blurred vision and amnesia. The player was then referred to an Accident and Emergency Department, closest to the field of play, for further evaluation by a physician. At the time of admission, symptoms of a painful and stiff neck had developed, as well as a feeling of pins and needles in the right hand, which was aggravated by movement. Following a clinical examination, an X-ray of the cervical spine was performed, which showed no abnormalities. A further clinical examination revealed no other neurological signs or symptoms. The patient was admitted overnight for observation. The following day, he was discharged as no deterioration in the clinical picture was noted. He was prescribed pain medication (Paracetamol) when necessary, provided with a soft collar and an information leaflet requesting him to seek medical attention if his symptoms deteriorated.

Five days after the initial trauma, the patient was evaluated by a physiotherapist and where he requested removal of the soft collar. However, clinical examination revealed a sensory loss at the C4–C5 dermatome of the right arm.

The patient was then referred to a sports physician, who confirmed the findings of the physiotherapist. Further examination using the Sideline Concussion Assessment Tool (SCAT) was performed. Symptoms noted included headache, difficulty concentrating, drowsiness, difficulty getting to sleep, balance disturbance, feelings of irritablity, nervousness, slowed down movement and feelings of fogginess, and body numbness, which totalled a score of 10.

Subsequently, a neuropsychological (NP) online examination (CogSport^®^) was performed, which resulted in a total score of 462 out of a possible 600, indicating an abnormality of neuropsychological functioning. Due to the ongoing neurological symptoms in the right arm, a Magnetic Resonance Imaging (MRI) of the cervical spine was requested, which showed a mild chronic bulge of the disc on the right C6–C7 level.

Unfortunately, the patient did not comply with the arranged follow-up visit and only presented again at the clinic on Day seventeen after injury. At this visit, he presented with symptoms of headache, vomiting, dizziness, sleepiness and a sensitivity to light. A C4–C5 dermatome sensory loss was still present in the right arm. Furthermore, weakness of handgrip strength and shoulder movement were also noted. There were still no clinical signs of raised intracranial pressure or structural intracranial pathology, specifically a fundoscopical examination was considered normal. The SCAT score was once again 10. A repeat online neuropsychological examination was conducted, which showed deterioration in test results (scoring 427 out of 600). The abnormal pattern of persisting symptoms raised suspicion for undiagnosed intracranial pathology, and therefore an immediate referral to a neurosurgeon was made.

The neurosurgical evaluation revealed a distressed and uncomfortable patient, clutching his neck and occipital region, with some non-dermatomal sensory loss bilaterally in the upper limbs. An initial suspicion of transverse sinus thrombosis was raised. It should be noted that the patient’s family confirmed a long history of recurrent, severe headaches suffered by this patient as a minor.

**Fig. 1:**
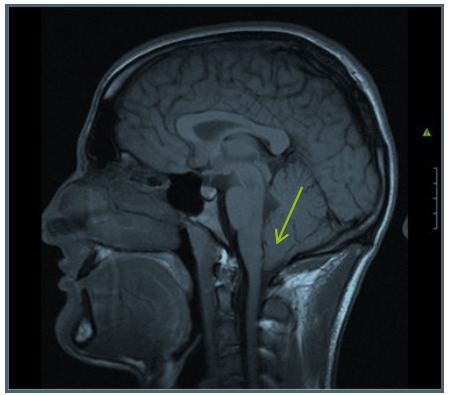
MRI of the brain (Day 17)

The MRI of the brain (Fig. 1) revealed low-lying cerebellar tonsils, with no hydrocephalus or syringomyelia. A diagnosis of a Chiari malformation, Type 1 (CM-I), was made. Immediate decompression surgery was performed by means of an enlargement of the foramen magnum and partial C1 laminectomy, followed by duraplasty. After a period of physical and cognitive rehabilitation, the patient resumed activities of daily living and completed his academic studies at university the following year. Furthermore, there was an uncomplicated course of events post-surgery.

## Discussion

In the acute setting, it can often be a challenge to differentiate SRC from potential life-threatening intracranial pathology, such as an intracranial haemorrhage or cerebellar herniation with compression of the tonsils due to a CM-I.

The Berlin Consensus Statement on concussion states that in a SRC “…*the impairment of neurological function resolves spontaneously and completely”*.^[1]^ This case illustrates the importance of a structured and repeated clinical assessment (including a thorough history, clinical examination and special investigations) where the SCAT and NP tests play an integral part in the monitoring of the athlete’s recovery and resolution of signs and symptoms over time. In this case, a deterioration of initial symptoms was noticed, as well as the onset of new symptoms, with an accompanying, unexpected deterioration of SCAT and NP testing results. This deviation in the expected pattern of resolution of symptoms led to the suspicion of further pathology. Eventually, a diagnosis of a CM-I, where classification is based on severity^[2]^, was made and the patient was managed accordingly.

The prevalence of a CM-I in the normal population varies from 0.2% to 3.6%.^[3]^ In this condition, the pathophysiological mechanisms of altered cerebrospinal fluid flow create a negative downward pressure gradient across the craniocervical junction, and, if combined with raised intracranial pressure, the presenting clinical symptoms may vary and might be persistent.^[4]^ Although most people with the CM-I condition remain asymptomatic, acute symptoms may develop either spontaneously or after trauma.^[5]^ In a study by Yarbrough et al. which investigated the acute onset of neurological symptoms in children with CM-I, half of all cases described were following minor head trauma. One of these cases was a football player who developed a paresthesia of the arm after a blow to the head during a flag football game.^[5]^

The safety of sports participation in patients with asymptomatic CM-I, especially in contact or collision sports, is still debated, considering the incidence of concussion in male football players is relatively low (1.08 concussions per 1000 athlete exposures, where one exposure is considered a single practice or game).^[6]^ A study by Meehan et al. describing 147 athletes with CM-I from different sporting codes who were followed over time, reported that not one sustained a catastrophic injury over the 3 year period.^[7]^ Similarly, a study by Strahle et al., which evaluated 503 CM-I patients (of which 328 participated in sports) over a 46 month period, reported no serious or catastrophic neurological injuries in the entire cohort.^[8]^ Thus it appears that the condition is benign without the presence of an inciting event. Although literature on this topic is limited, most authors suggest a case-by-case evaluation, with permission to take part in contact sports, following counselling from a neurosurgeon on the possible risks.

## Conclusion

This case report illustrates the importance of a structured clinical follow-up of concussion in sports. It is important for medical staff to recognise sequential patterns in developing symptoms which could indicate possible intracranial pathology. The use of the SCAT and importance of NP testing in the sequential follow-ups raised suspicion of a complicated process of recovery from SRC in this particular case. Whilst this patient presented with evolving symptomatology and eventually required surgical intervention, literature suggests that overall sports participation in patients with asymptomatic CM-I is a low risk for morbidity and mortality, even for contact and collision sports, including football.
